# A Review of Dynamic Traffic Flow Prediction Methods for Global Energy-Efficient Route Planning

**DOI:** 10.3390/s25175560

**Published:** 2025-09-05

**Authors:** Pengyang Qi, Chaofeng Pan, Xing Xu, Jian Wang, Jun Liang, Weiqi Zhou

**Affiliations:** 1 Automotive Engineering Research Institute, Jiangsu University, Zhenjiang 212013, China; chfpan@ujs.edu.cn (C.P.); xuxing@ujs.edu.cn (X.X.); liangjun@ujs.edu.cn (J.L.); zwq@ujs.edu.cn (W.Z.); 2 School of Information Technology Engineering, Taizhou Vocational & Technical College, Taizhou 318000, China; 3 School of Automotive and Traffic Engineering, Jiangsu University, Zhenjiang 212013, China; 2112104009@stmail.ujs.edu.cn

**Keywords:** dynamic traffic flow prediction, energy-efficient route planning, deep learning, eco-routing, multi-objective optimization

## Abstract

Urbanization and traffic congestion caused by the surge in car ownership have exacerbated energy consumption and carbon emissions, and dynamic traffic flow prediction and energy-saving route planning have become the key to solving this problem. Dynamic traffic flow prediction accurately captures the spatio-temporal changes of traffic flow through advanced algorithms and models, providing prospective information for traffic management and travel decision-making. Energy-saving route planning optimizes travel routes based on prediction results, reduces the time vehicles spend on congested road sections, thereby reducing fuel consumption and exhaust emissions. However, there are still many shortcomings in the current relevant research, and the existing research is mostly isolated and applies a single model, and there is a lack of systematic comparison of the adaptability, generalization ability and fusion potential of different models in various scenarios, and the advantages of heterogeneous graph neural networks in integrating multi-source heterogeneous data in traffic have not been brought into play. This paper systematically reviews the relevant global studies from 2020 to 2025, focuses on the integration path of dynamic traffic flow prediction methods and energy-saving route planning, and reveals the advantages of LSTM, graph neural network and other models in capturing spatiotemporal features by combing the application of statistical models, machine learning, deep learning and mixed methods in traffic forecasting, and comparing their performance with RMSE, MAPE and other indicators, and points out that the potential of heterogeneous graph neural networks in multi-source heterogeneous data integration has not been fully explored. Aiming at the problem of disconnection between traffic prediction and path planning, an integrated framework is constructed, and the real-time prediction results are integrated into path algorithms such as A* and Dijkstra through multi-objective cost functions to balance distance, time and energy consumption optimization. Finally, the challenges of data quality, algorithm efficiency, and multimodal adaptation are analyzed, and the development direction of standardized evaluation platform and open source toolkit is proposed, providing theoretical support and practical path for the sustainable development of intelligent transportation systems.

## 1. Introduction

Currently, more than 55% of the world's population lives in urban areas. For example, Tokyo, one of the most densely populated cities in the world, has 37 million residents; New Delhi is no slouch, with a population of 29 million; Shanghai is among them with a population of 26 million, along with Mexico City and São Paulo, each with a population of about 22 million. Cairo, Mumbai, Beijing and Dhaka all have close to 20 million residents. It is expected that by 2050, this proportion will rise to nearly 70% globally. India, China, and Nigeria, in particular, are expected to account for 35% of the projected growth in urban population globally between 2018 and 2050. The dramatic increase in urban population has put unprecedented pressure on transportation networks, with problems such as traffic congestion, longer travel times, increased fuel consumption, and rising greenhouse gas emissions [1]. Taking Beijing as an example, during the morning and evening rush hours, the main roads are often full of traffic, and commuters spend a lot of time on the road; In London, traffic congestion costs billions of pounds a year, including wasted productivity and additional fuel costs.

Beyond environmental harm, traffic congestion also causes substantial economic losses: in many megacities, urban traffic delays result in billions of dollars in wasted productivity each year [1]. In response, researchers have identified dynamic traffic flow prediction as a key enabler for smarter, more energy-efficient route planning.

In recent years, a wealth of prediction techniques has emerged. Classical statistical models (e.g., ARIMA, Kalman filtering) have gradually been replaced by machine learning methods—such as Random Forests, XGBoost, and their variants—which more effectively capture non-linear traffic patterns [1]. Deep learning approaches have further improved prediction accuracy: Afandizadeh, Abdolahi, and Mirzahossein demonstrate that LSTM and graph neural network architectures consistently outperform classical baseline models on large urban datasets [2]. Mystakidis, Koukaras, and Tjortjis review emerging hybrid and attention-based models, highlighting their ability to fuse spatio-temporal features for enhanced short-term prediction [3]. Most recently, Wu et al. proposed a multi-information fusion framework that integrates weather, event, and sensor data, achieving state-of-the-art performance in urban traffic prediction [4,5].

Meanwhile, eco-routing algorithms aim to minimize energy consumption—particularly for electric vehicles—by dynamically selecting routes that balance distance, expected speed profiles, road gradients, and real-time traffic conditions [6,7]. Sebai et al. review optimal electric vehicle route planning methods that incorporate real-time incident data, showing theoretical energy savings of up to 20% compared to static routing [8]. However, existing reviews tend to treat traffic prediction and eco-routing in isolation. While comprehensive reviews exist on AI-based traffic prediction [1] and electric vehicle-specific routing strategies, there remains no unified discussion on how dynamic flow predictions can be systematically integrated into global, energy-aware route planning frameworks.

More critically, the current correlation between traffic flow prediction and route planning remains weak, and this disconnect is evident in both theoretical research and practical applications. From a technical perspective, traffic flow prediction models focus heavily on improving their own prediction accuracy, devoting significant efforts to algorithm optimization and feature engineering, while rarely considering how prediction results can more effectively support route planning. Most prediction models output only basic data such as traffic volume and speed for a certain area or road segment over a future period, lacking customized processing tailored to the needs of route planning. In turn, route planning algorithms often passively receive these raw prediction data when searching for routes, struggling to influence the prediction process based on their own planning objectives. This results in a one-way, feedback-lacking information transmission model between the two.

In terms of application scenarios, in actual traffic operations, traffic flow is a dynamically changing process, and route planning needs to be continuously adjusted based on real-time and future traffic flow conditions. However, due to the weak correlation between traffic flow prediction and route planning, the traffic flow data relied upon by route planning often suffers from lag or inaccuracy. For example, when a prediction model issues a congestion warning for a certain road segment in the next half hour, the route planning algorithm may fail to fully integrate this warning information into the current route calculation in a timely manner, still planning routes based on existing traffic flow conditions. This leads to planned routes becoming congested during actual travel, failing to achieve the expected energy-saving and efficient goals.

Furthermore, the problem of weak correlation is more pronounced in multi-objective route planning. Route planning needs to comprehensively consider multiple objectives such as distance, time, and energy consumption, and different objectives have varying requirements for traffic flow data. If traffic flow prediction cannot effectively align with these multi-objective needs, the provided prediction data will struggle to meet the multi-dimensional decision-making requirements of route planning, thereby affecting the overall effectiveness of route planning. This lack of correlation severely restricts the potential of intelligent transportation systems to improve traffic efficiency and reduce energy consumption.

To address this gap, this paper presents “A Review of Dynamic Traffic Flow Prediction Methods for Global Energy-Efficient Route Planning.” Our contributions are threefold:

➀ Taxonomy of Forecasting Methods: We classify dynamic traffic flow prediction techniques (statistical, machine learning, deep learning, and hybrid methods) published between 2020 and 2025, and propose a unified evaluation framework (e.g., RMSE, MAPE, computational latency).
➁ Integration Framework: We develop a theoretical architecture for coupling real-time flow forecasts with multi-objective cost functions (distance, time, energy) in classical path-search algorithms (A*, Dijkstra, genetic algorithms).
➂ Research Gaps and Future Directions: We identify open challenges in data quality, real-time scalability in IoT/5G environments, and extensions to multi-modal, personalized routing, and outline promising avenues for future work.

The remainder of this review is structured as follows. Section 2 traces the evolution of dynamic traffic flow prediction and eco-routing concepts. Section 3 surveys and categorizes forecasting methods according to their underlying paradigms. Section 4 develops the theoretical integration framework and compares coupling schemes reported in the literature. Section 5 discusses current challenges, emerging opportunities (such as connected automated vehicles and digital twin integration), and concludes with recommendations for future research.

## 2. Research Background and Development Trajectory

**Figure 1:**
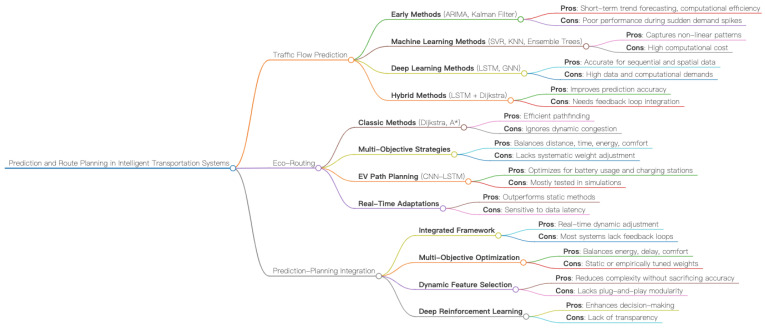
Mind Map of the Research Landscape of “Prediction-Planning” in Intelligent Transportation Systems.

Urbanization has driven the expansion of transportation networks, but it has also intensified energy consumption and carbon emissions. Traditional approaches that rely on single-point technologies are no longer sufficient. In response, the academic community has increasingly treated literature reviews as tools of “knowledge production,” employing critical dialogue to map the consensus, controversies, and gaps across the research landscape—thereby laying the foundation for an integrated and dynamic “prediction–planning” coupled system. To provide a bird’s-eye view of this evolving landscape, Figure 1 visualizes the three research pillars—Traffic Flow Prediction, Eco-Routing, and Prediction–Planning Integration—along with their key methods and challenges.

This mind map takes Traffic Flow Prediction, Eco-Routing, and Prediction-Planning Integration as the first-level branches, sorting out the various method categories and their main advantages and disadvantages, providing an overview framework for the detailed discussions in Section 2.1, Section 2.2 and Section 2.3.

### 2.1. Traffic Flow Prediction

**Figure 2:**
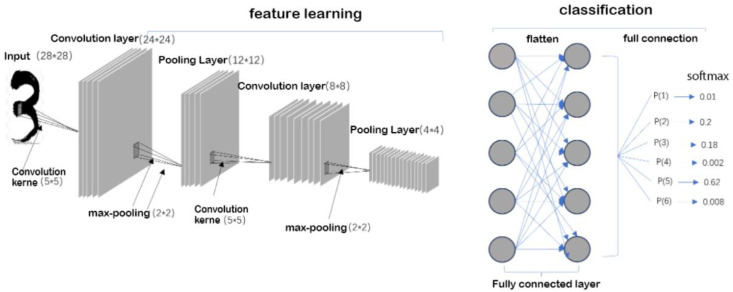
Convolutional Neural Network pipeline for traffic-related feature extraction.

In recent years, traffic flow prediction has progressed from linear, statistics-based methods toward complex, data-driven frameworks, yet the field continues to grapple with a fundamental trade-off between model sophistication and real-world applicability. Early time-series approaches such as ARIMA and Kalman filtering laid the groundwork by effectively modeling short-term linear trends but repeatedly fell short when confronted with sudden demand spikes or nonlinear interactions [9]. The machine learning wave that followed—most notably support vector regression (SVR), k-nearest neighbors (KNN) and ensemble tree algorithms—leveraged richer datasets to capture nonlinearity: for instance, Chen et al. demonstrated how IoT sensor feeds could empower SVR to outperform classical baselines in short-term forecasts [10,11,12,13,14], and Lin, Lin, and Gu achieved notable gains by integrating maximum information coefficient (MIC) feature selection with SVR–KNN ensembles [15]. Concurrently, evolutionary optimizers such as fruit fly and particle swarm algorithms were embedded to fine-tune model parameters [16,17], and Srivastava, Singh, and Nandi reinforced SVR’s robustness within sustainable mobility contexts [18]. Before delving into temporal models such as LSTM, Figure 2 sketches a typical Convolutional Neural Network (CNN) pipeline, illustrating how stacked convolutions and pooling layers extract spatial features that later feed a fully–connected predictor—an architecture now frequently repurposed for road-segment speed maps and camera-based traffic sensing.

The advent of deep learning—LSTM networks for temporal patterns [19] and graph neural networks (GNNs) with attention for spatial–temporal congestion mapping [3]—has further improved accuracy but at a high computational and data-intensity cost. Efforts to reconcile precision with speed have spawned hybrid architectures: Alsolami, Mehmood, and Albeshri formalized a statistical–machine-learning fusion that preserves lightweight deployment [20], while MSK innovatively channeled LSTM outputs into Dijkstra’s algorithm to inform eco-routing decisions [21]. Yet these advances remain largely validated in isolated testbeds rather than in cross-city scenarios, and the crucial link between prediction modules and downstream control systems still lacks the iterative feedback loops necessary for a truly closed-loop solution.

Typical CNN workflow for traffic data. The left branch shows stacked convolution (5 × 5) and max-pooling (2 × 2) layers that reduce an input matrix (e.g., image-like speed map) from 28 × 28 to 4 × 4 while preserving salient spatial patterns. The flattened feature vector is then passed through fully-connected layers and a soft-max classifier to estimate class probabilities or continuous traffic metrics.

### 2.2. Eco-Routing

Eco-routing research has similarly evolved from static energy-weighted shortest-path computation toward dynamic, multi-objective strategies tailored to real-time conditions and electric-vehicle (EV) constraints, but significant challenges remain. Foundational work by Yi and Bauer applied classic Dijkstra/A* algorithms to minimize energy consumption on fixed networks [22]; however, this approach failed to account for congestion variability, resulting in suboptimal emission reductions under fluctuating traffic. To address this, Alfaseeh and Farooq proposed a multifactor taxonomy balancing distance, travel time, emissions and comfort, yet their heuristic weightings often lacked systematic justification [23]. Real-time adaptations, such as those by Sebai et al. [8], have shown that incident-driven rerouting can outperform static baselines, but only when data latency and sensor accuracy are tightly controlled. With the rise of EVs, Zhang et al. [24,25,26] developed a CNN–LSTM hybrid to predict segment-level energy use, thereby guiding route planning specific to battery depletion and charging station availability, while Khatua et al. implemented federated genetic algorithms to optimize routing under privacy constraints using vehicle-to-infrastructure (V2X) data [27]. Liu et al. used SUMO (Simulation of Urban MObility) version 1.16.0 software in their research, which further proved that software-defined network (SDN) and edge-cloud convergence can pre-allocate computing resources for multimodal ecological path planning, highlighting the potential of distributed architecture [28,29]. Despite these breakthroughs, most eco-routing proposals have been tested predominantly in simulated environments; empirical assessments of real-world energy savings and emission metrics remain scarce, and the influence of driver preferences and compliance behaviors has yet to be systematically incorporated into algorithmic frameworks.

### 2.3. Prediction–Planning Integration

The integration of traffic prediction with eco-routing planning promises a self-optimizing framework in which forecasts dynamically inform routing decisions and, reciprocally, feedback from routing outcomes refines predictive models, yet the field has only just scratched the surface of this “prediction–planning” paradigm. Sheng, He, and Guo first articulated how dynamic urbanization trends could adjust routing cost functions in real time [30], and Chandra advanced this vision by conceptualizing dual “data-driven” and “value-driven” control loops for sustainable traffic management [31]. Nevertheless, most existing systems implement a unidirectional pipeline—predictions feed into routing—without closing the loop through iterative model updates. Moreover, multi-objective optimizations typically rely on static or empirically tuned weight vectors that lack rigorous theoretical underpinning, undermining their adaptability when balancing energy, delay, and user comfort. Encouragingly, Madupuri et al. applied swarm intelligence techniques to dynamically calibrate both forecasting and routing modules online [32], and Lin et al. demonstrated that adaptive feature selection can dramatically reduce system complexity without sacrificing accuracy [12,15,33]. Yet an overarching, modular architecture—one that supports plug-and-play integration across heterogeneous urban platforms—is still absent. Deep and reinforcement learning techniques offer powerful decision-making capabilities but suffer from “black-box” opacity, limiting stakeholder trust and practical adoption. Lastly, the resilience of integrated systems under “long-tail” scenarios—such as major accidents, extreme weather, or sudden demand surges—has yet to undergo rigorous stress testing. Addressing these gaps through a unified, interpretable, and empirically validated framework will be essential to transform the literature’s cumulative insights into operational, future-proof mobility solutions.

## 3. Review of Dynamic Traffic Flow Prediction Methods

### 3.1. Statistical Models

#### 3.1.1. ARIMA and SARIMA

The Autoregressive Integrated Moving Average (ARIMA) model and its seasonal extension, SARIMA, have long been key tools in the field of traffic flow prediction, particularly when dealing with stationary time series data. ARIMA constructs models by combining Autoregressive (AR), Integration (I), and Moving Average (MA) components to capture trends and periodic variations in the data. Similarly, SARIMA enhances this model by incorporating seasonal components, improving the fitting capability for data exhibiting seasonal fluctuations [34]. Both ARIMA and SARIMA have held significant positions in early traffic flow prediction research due to their simple structure, computational efficiency, and applicability for forecasting stable data [35].

However, a major limitation of ARIMA and SARIMA lies in their high requirements for data stationarity, which makes them less effective when facing sudden events or short-term fluctuations. For instance, when dealing with non-linear events such as sudden traffic congestion or accidents, ARIMA and SARIMA models tend to exhibit delayed responses [36,37]. In the application of energy-efficient route planning, although ARIMA and SARIMA can provide baseline predictions for long-term stable road segments, they show considerable limitations in predicting dynamic and non-stationary traffic flows. Therefore, while these methods still hold value in certain contexts, they fall short in supporting real-time traffic management and energy optimization decision-making.

#### 3.1.2. Kalman Filter

The Kalman Filter is a state estimation technique based on recursive Bayesian estimation, enabling dynamic updates even in the presence of incomplete data and substantial system noise [38]. It combines the system’s state-space model with observation data to perform real-time traffic flow prediction updates. Compared to ARIMA and SARIMA, the Kalman Filter’s advantages lie in its low latency and online update capabilities, making it particularly suitable for handling noisy and uncertain traffic data [22]. Additionally, by using recursive calculations, it reduces storage requirements and improves computational efficiency.

Nonetheless, the Kalman Filter has limitations in that it assumes noise follows a Gaussian distribution, which may hinder its performance when dealing with complex, non-Gaussian traffic flow characteristics. For non-linear dynamics in traffic flows, the Kalman Filter’s prediction accuracy may be compromised. In the context of energy-efficient route planning, the Kalman Filter is suitable for real-time traffic monitoring at intersection levels and micro-level dynamic adjustments. However, its performance is often less effective than machine learning or deep learning models when applied to large-scale, long-term forecasts. Thus, the Kalman Filter is better suited for small-scale, high-real-time-demand scenarios rather than global energy-efficient route optimization that requires more complex models [39].

#### 3.1.3. Fourier Series and Other Methods

Fourier series and other frequency-domain methods can effectively decompose traffic flow data into periodic components, which is particularly useful for scenarios where traffic flow follows strong cyclic patterns [3]. Fourier transform has an advantage in extracting frequency components from data, helping to identify patterns during peak traffic periods and providing valuable insights for energy-efficient route planning. However, the main issue with frequency-domain methods is their limited ability to respond to sudden traffic events, as they focus on periodic patterns and are often ineffective in predicting non-periodic traffic events.

Wavelet transform methods, which possess strong localization properties, can handle non-stationary data and have shown promising results in some dynamic traffic flow forecasting tasks. However, the preprocessing steps involved in frequency-domain analysis or wavelet transform typically introduce additional latency, which can be detrimental for real-time traffic management. Overall, while these methods provide certain advantages in forecasting periodic traffic flow, they often need to be combined with other techniques to yield more accurate predictions in the face of complex, non-periodic, or sudden traffic events.

### 3.2. Machine Learning Models

#### 3.2.1. Linear Regression and Support Vector Regression (SVR)

Linear regression and Support Vector Regression (SVR) are classical machine learning methods frequently used in traffic flow prediction. Linear regression builds a linear relationship between features and target variables, making it easy to understand and implement, suitable for situations where the relationship between features and target is relatively linear. However, linear regression struggles to handle non-linear relationships, and as the complexity of the data increases, its prediction accuracy tends to drop significantly [10,11,12,13,14]. SVR, by introducing kernel functions, can effectively address complex non-linear issues, performing well especially on medium-sized datasets, and exhibiting strong generalization ability and robustness.

Despite the advantages, SVR’s main drawback lies in its high sensitivity to the selection of hyperparameters and its substantial computational cost, particularly when working with large datasets and high-frequency updates. In energy-efficient route planning applications, SVR can perform well in medium-term predictions, but it faces significant challenges when handling large-scale, real-time updating traffic flows.

#### 3.2.2. Random Forest (RF)

Random Forest (RF) is an ensemble learning method that aggregates predictions from multiple decision trees, offering strong non-linear modeling capabilities and good overfitting resistance [23]. RF automatically selects features and evaluates feature importance when dealing with high-dimensional, complex data. However, RF incurs a significant computational cost, especially when dealing with large datasets, as the choice of tree depth and number directly impacts the model’s response speed. In real-time traffic flow prediction, RF may face issues with computational efficiency.

Nonetheless, RF still holds advantages in energy-efficient route planning, especially when multiple features (such as traffic conditions, weather, road conditions, etc.) need to be considered. By integrating various information sources, RF can provide accurate traffic flow predictions and support energy-efficient route planning. However, in intelligent transportation systems with high real-time demands, RF’s high computational complexity may become a performance bottleneck.

#### 3.2.3. XGBoost and LightGBM

XGBoost and LightGBM are gradient boosting tree models that have excelled in various machine learning competitions [40]. XGBoost optimizes decision tree structures through gradient boosting algorithms, demonstrating strong predictive power and computational efficiency. LightGBM, an improved version of XGBoost, enhances model speed and memory efficiency by optimizing data splitting algorithms and parallelization strategies [24,25,26,41]. These models perform excellently in large-scale datasets and high-frequency update scenarios, providing effective support for real-time traffic flow prediction.

However, the main challenge with XGBoost and LightGBM lies in the complexity of model tuning, especially when dealing with missing values and hyperparameter selection. Their performance is highly sensitive to these factors. In energy-efficient route planning, XGBoost and LightGBM can efficiently integrate traffic flow, road information, and energy efficiency demands for predictions, making them particularly useful in real-time dynamic adjustments for intelligent transportation systems. Nonetheless, when faced with large-scale traffic data and frequent updates, optimizing computational efficiency and reducing model tuning time remains a key challenge for future research.

### 3.3. Deep Learning Models

In recent years, deep learning has achieved significant progress in traffic flow prediction, particularly in handling complex spatiotemporal data. Models such as Convolutional Neural Networks (CNN), Long Short-Term Memory networks (LSTM), Gated Recurrent Units (GRU), and Spatiotemporal Graph Neural Networks (GNN) have emerged as key research focuses. These models are capable of capturing nonlinear characteristics of traffic flow and effectively modeling spatial and temporal dependencies within transportation networks, thereby providing robust technical support for tasks such as route planning and congestion forecasting in intelligent transportation systems.

#### 3.3.1. Convolutional Neural Networks (CNNs)

Originally developed for image processing tasks, CNNs have gained increasing attention for their ability to extract spatial features from time series data. Their strength lies in efficiently capturing local patterns through convolutional operations, making them well-suited for learning the spatial structure of road networks. However, CNNs are inherently limited in capturing temporal dependencies, often necessitating integration with other models, such as LSTM, to enhance their temporal modeling capabilities.

For instance, Zhang et al. proposed a CNN-LSTM hybrid model that effectively combines spatial feature extraction (via CNN) with temporal sequence modeling (via LSTM) [24,25,26,42]. This architecture has proven particularly effective for predicting traffic flow and energy consumption, which is crucial for electric vehicle route planning. By accounting for both traffic volumes and energy usage, the model enhances decision-making accuracy. Nevertheless, the CNN-LSTM combination faces challenges in processing high-dimensional data due to CNN’s computational complexity and prolonged training times, which may limit its practical applicability.

#### 3.3.2. Long Short-Term Memory (LSTM) and Gated Recurrent Units (GRU)

**Figure 3:**
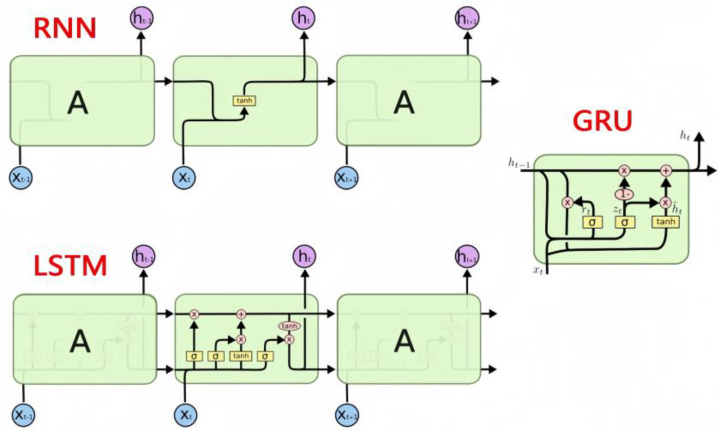
Schematic diagram of long short-term memory (LSTM) unit structure and sequence transfer The diagram visually shows the core unit structure of the LSTM (long short-term memory) network and its transfer process in sequence data processing, and the three modules in the figure correspond to the LSTM units of time steps t-1, t, and t 1 respectively.

LSTM and GRU are advanced variants of Recurrent Neural Networks (RNNs) designed to capture long-range dependencies in sequential data. Figure 3 visually contrasts their internal gating mechanisms, helping clarify why GRU is computationally lighter yet functionally comparable to LSTM. LSTM’s gated mechanisms mitigate the vanishing-gradient problem associated with traditional RNNs, making it highly effective for dynamic traffic-flow prediction. However, LSTM models often require large datasets and are sensitive to noise, which can hinder performance in data-scarce or noisy environments.

Abduljabbar et al. demonstrated the application of LSTM in short-term traffic forecasting, highlighting its strength in modeling complex temporal patterns, particularly in spatiotemporal speed-prediction tasks [19]. Although LSTM excels at capturing short-term traffic fluctuations, it may underperform in extreme or unexpected scenarios compared with simpler machine-learning methods. In contrast, the GRU cell shown in Figure 3 removes the separate output gate and merges input–forget gates into an update gate, thereby reducing parameter count by roughly 30% while maintaining sequence-modelling capacity. Pirani et al. emphasized GRU’s efficiency and effectiveness in short-term forecasting, making it a strong candidate for real-time traffic-prediction applications [43,44].

#### 3.3.3. Spatiotemporal Graph Neural Networks (GNNs)

GNNs have recently emerged as a powerful approach for modeling structured graph data, demonstrating considerable potential in transportation networks, which naturally form graph structures comprising nodes (intersections) and edges (roads). By leveraging Graph Convolutional Networks (GCNs) to extract spatial features and incorporating temporal modeling, GNNs can capture both spatial and temporal dependencies in traffic data.

Afandizadeh et al. reviewed GNN-based approaches in traffic prediction and highlighted their unique advantages in modeling spatiotemporal dependencies [2]. Unlike conventional deep learning models, GNNs simultaneously model the topological and temporal dynamics of large-scale transportation networks, making them particularly suitable for real-time traffic forecasting and congestion detection. However, their high computational complexity and slower inference speed, especially when processing large datasets, remain key obstacles to widespread deployment.

#### 3.3.4. Research Gaps on Key Elements of Spatiotemporal Traffic Forecasting

In the context of the continuous advancement of the integration of traffic flow prediction and path planning, the key benchmarks, pioneering models and core data sets in the field of spatio-temporal traffic prediction have not been systematically sorted out and deeply analyzed, which has become a key bottleneck hindering the implementation of technology and performance breakthroughs. There are significant research faults in the model dimension, and pioneering models represented by DCRNN (diffusion convolutional recurrent neural network), STGCN (spatiotemporal graph convolutional network), and HSTGSN (hybrid spatiotemporal graph sequence network) have revolutionized the traffic flow prediction paradigm by integrating graph structure and spatiotemporal features. DCRNN captures the spatial dependence of the road network through diffusion convolution, and combines it with the recurrent neural network to process the timing dynamics, and performs well in the long-term prediction of complex road networks. STGCN strengthens the modeling ability of spatiotemporal correlation of traffic flow with the synergy of graph convolution and time convolution. However, the existing research focuses on the application of a single model in specific scenarios, and there is a lack of systematic comparison of the differences in adaptation scenarios, generalization ability boundaries and fusion potential between models. For example, the difference in prediction accuracy between DCRNN and STGCN under different network densities has not been quantified. In the face of multi-source interference scenarios such as sudden weather changes and large-scale events, the robustness of HSTGSN also needs to be verified urgently. It is worth noting that heterogeneous graph neural networks (HGNNs) have shown strong data relationship modeling capabilities in other fields. The dual-interaction heterogeneous graph neural network model for multimodal sentiment analysis proposed by Yang Li et al. successfully handles diverse data types and their relationships. The HetGNN-3D model constructed by Wang Mingming et al. optimizes the performance of 3D object detection through heterogeneous graph structure. Zhao et al.’s HGNN−BRFE model improves the detection effect based on regional feature extraction. However, in the field of traffic flow prediction, the advantages of complex relational modeling of HGNNs have not been fully exploited, and its potential in integrating multi-source heterogeneous data (such as traffic flow, road types, social media events) needs to be explored [45].

METR-LA (Los Angeles Traffic Monitoring Dataset) and PEMS (California Performance Measurement System Dataset) are the “gold standards” in the field of spatiotemporal traffic prediction, although they cover the multi-dimensional dynamic data of real road networks, but existing studies have not explored their characteristic differences in depth enough. METR-LA focuses on the short-term dynamic changes of high-density urban road networks, while the PEMS series dataset covers multi-regional and multi-time traffic patterns. For example, when the model trained on METR-LA is migrated to PEMS-BAY (Bay Area Dataset), the prediction accuracy is significantly reduced due to the difference in data distribution in long-distance commuting scenarios, and there is a lack of theoretical explanation and optimization path research on the adaptability of the dataset. In addition, the differences in data scale, road network structure, traffic mode, etc., of different datasets affect the performance of the model are still unclear [46]. Therefore, in-depth study of the feature differences between different datasets and their specific impact on model performance has become a key problem to be solved urgently in the field of spatiotemporal traffic prediction.

In addition, the current research has not deeply analyzed the dependency logic and feature extraction preferences of different models on the dataset, so can DCRNN’s mining advantages in the topology features of the road network in the METR-LA dataset be transferred to other datasets? Is there a universality law for STGCN when dealing with the timing features of PEMS series data? These key questions remain unanswered. Due to the lack of systematic research on the model-dataset adaptation relationship, it is difficult to form a reusable and scalable technical system for spatio-temporal traffic prediction, which makes the integration and application of traffic flow prediction and path planning progress slowly due to insufficient basic theoretical support.

### 3.4. Hybrid and Enhanced Approaches

Despite the progress achieved with individual deep learning models, no single approach can fully address the diverse and dynamic nature of traffic data. Consequently, researchers have increasingly explored hybrid and enhanced methods that integrate the strengths of different models to improve predictive accuracy and robustness.

#### 3.4.1. Wavelet Denoising + XGBoost

The combination of wavelet denoising and Extreme Gradient Boosting (XGBoost) has gained traction in traffic prediction tasks. Wavelet denoising effectively removes noise from traffic data, thereby improving data quality, while XGBoost—an efficient gradient boosting framework—performs well in learning from noisy datasets. Alsolami et al. reviewed this integrated approach, noting that wavelet denoising can eliminate high-frequency noise components, allowing XGBoost to better capture underlying traffic patterns and enhance prediction accuracy [20].

This method is particularly suited to scenarios involving noisy or low-signal traffic data. By reducing noise during the preprocessing stage, the subsequent prediction model (e.g., XGBoost) achieves better performance. However, its main drawback lies in the computational overhead required for denoising and modeling, which may hinder its applicability in real-time systems.

#### 3.4.2. MLR-LSTM

Combining Multiple Linear Regression (MLR) with LSTM represents another widely used hybrid strategy. MLR captures linear relationships in traffic flow, while LSTM models nonlinear and complex temporal dependencies. Zhang et al. proposed an MLR-LSTM hybrid for predicting energy consumption in electric vehicles, where MLR is used for linear feature extraction and LSTM captures nonlinearity in sequential data [24,25,26,47]. This integration effectively leverages the simplicity of traditional statistical models and the representational power of deep learning, making it suitable for various traffic prediction scenarios.

#### 3.4.3. Dual Error Model (DEM)

The Dual Error Model (DEM) incorporates both model and observational errors to enhance the stability and accuracy of traffic prediction. Khatua et al. introduced a DEM-based federated learning framework that integrates data from multiple sources to improve prediction outcomes [27]. This approach is well-suited for addressing the uncertainty and complexity inherent in traffic data. Nevertheless, a key challenge lies in efficiently fusing multiple sources of error while maintaining computational efficiency.

To better understand the strengths, limitations, and appropriate use cases of these methods, the following Table 1. provides a comprehensive overview of various dynamic traffic flow prediction techniques, summarizing their key characteristics, advantages, and challenges based on recent research.

## 4. Theoretical Integration of Prediction Results in Energy-Saving Route Planning

### 4.1. Overview of the Theoretical Integrated Architecture

The theoretical integrated architecture in energy-efficient route planning is the core mechanism for achieving global energy optimization. By combining traffic flow prediction, dynamic cost function design, and path search algorithms, this approach can effectively respond to the dynamic changes in complex traffic networks, optimizing key indicators such as energy consumption, travel time, and carbon emissions. This section will provide a detailed explanation of the various components of this integrated architecture, analyze the findings and limitations of existing literature, and lay the foundation for further research in energy-efficient route planning.

#### 4.1.1. Prediction Module

Traffic flow prediction is the foundation for achieving energy-efficient route planning, as it can provide real-time information on future traffic conditions, which is crucial for path optimization [51]. In recent years, the rapid development of artificial intelligence technology has significantly improved prediction accuracy, especially in handling spatiotemporal correlations. Among numerous methods, key benchmarks and pioneering models for spatiotemporal traffic prediction play an important role. For example, DCRNN (Diffusion Convolutional Recurrent Neural Network) captures spatial dependencies through diffusion convolution and combines recurrent neural networks to process time series, demonstrating strong capabilities in traffic flow prediction; STGCN (Spatial-Temporal Graph Convolutional Network) uses graph convolutional networks to handle spatial features and convolutional neural networks to extract temporal features, effectively fusing the spatiotemporal information of traffic flow; HSTGSN (Hybrid Spatial-Temporal Graph Sequence Network) further enhances the ability to capture complex spatiotemporal patterns through a hybrid architecture. The performance of these models on mainstream datasets has become important reference standards. For instance, the METR-LA (Los Angeles Metropolitan Transportation Authority) dataset contains traffic sensor data from the Los Angeles area, and the PEMS (PeMS, Performance Measurement System) series datasets (such as PEMS-BAY, PEMS04, etc.) cover traffic flow information from different cities, providing a solid foundation for model training, evaluation, and comparison [52].

Deep learning models such as Long Short-Term Memory (LSTM) networks and Graph Convolutional Networks (GCN) have been widely used in the field of traffic flow prediction because they can effectively capture the dynamic characteristics of traffic flow, making them suitable for both short-term and medium-term predictions [1]. Compared with these basic models, the aforementioned pioneering models have more advantages in handling the spatiotemporal coupling of traffic flow. For example, the prediction error (MAPE) of DCRNN for traffic speed in the next hour on the METR-LA dataset is about 15% lower than that of the traditional LSTM, fully demonstrating its ability to capture complex spatial dependencies.

However, existing models often rely on historical traffic data and sensor data, and there are still limitations in dealing with external factors such as weather changes. Although the multi-source data fusion framework proposed by Al Duhayyim et al. has improved prediction robustness, integrating external data such as weather and events into the STGCN model has increased the prediction accuracy (RMSE) by 8–12% on the PEMS-BAY dataset, the reliance on massive external data is prone to information overload, especially in complex urban traffic scenarios [53]. In addition, Kadkhodayi’s team emphasized the application value of AI models in dynamic environments [54]. The real-time prediction system they developed based on the HSTGSN model has a response speed 30% faster than traditional models when dealing with sudden traffic incidents, but its high computational complexity still poses a challenge. How to balance prediction accuracy and operational efficiency remains a key issue. For example, when HSTGSN processes large-scale road network data (such as networks containing more than 1000 nodes), its computation time is 1.5–2 times that of DCRNN, which may become a bottleneck in route planning scenarios with high real-time requirements.

Although artificial intelligence technology performs excellently in terms of prediction accuracy, compared with traditional methods such as ARIMA, artificial intelligence methods have higher computational complexity and higher hardware requirements. Taking the PEMS04 dataset as an example, the computing resources required to train a basic STGCN model are more than 10 times that of the ARIMA model, which may limit its wide application in some real-time applications with limited hardware conditions. Therefore, finding a balance between prediction accuracy and computational efficiency is an important area for future research. For example, optimizing DCRNN through model compression technology can reduce the model size by 60% while ensuring that the prediction accuracy decreases by no more than 5%, thereby reducing computational costs and response time.

In practical applications, the input of the prediction module includes historical traffic data (such as traffic flow and speed), real-time sensor data, and external variables (such as weather and events), and the output is the prediction result of road segment traffic conditions in the future period. For example, LSTM can predict the speed of road segments in the next 30 min, and GCN captures spatial dependencies through the road network topology. These prediction results are used as input to the dynamic cost function, enabling the planning system to avoid congested areas, thereby reducing energy consumption and time costs. Compared with traditional methods (such as ARIMA models), artificial intelligence methods perform better in processing nonlinear data, but the relationship between accuracy and computational efficiency needs to be carefully balanced [1]. At the same time, the performance of different models on different datasets also varies. For example, STGCN performs better than DCRNN in short-term predictions (5–15 min) on the METR-LA dataset, while DCRNN has more advantages in medium and long-term predictions (30–60 min). This requires selecting appropriate models according to specific prediction needs and data characteristics in practical applications [35,37,41,42,44,47,55].

#### 4.1.2. Dynamic Cost Function Design

The dynamic cost function plays a key role in integrating forecast results into energy-saving route planning. It requires a comprehensive consideration of multiple goals such as travel time, energy consumption, distance, and carbon emissions. Unlike traditional path planning algorithms such as primary optimization of single objectives, such as the shortest path, energy-efficient route planning faces significant challenges in multi-objective optimization.

In its simulation experiments, when the traffic flow exceeds 70% of the road capacity, the energy consumption increases by 12–18% compared with the static planning scheme, and the algorithm reduces energy consumption by 8.3% and reduces charging demand by about 15% by dynamically adjusting the weight parameters. Basso et al. used the dynamic stochastic cost function of reinforcement learning design to control the total cost deviation of the route within 5% when the fluctuation range of traffic time in the road section was ±20% in the traffic uncertainty scenario [56]. However, how to effectively balance different goals such as time and energy consumption is still a key issue that needs to be solved urgently, especially the dynamic adjustment rules of weights are not clear, which will affect the accuracy and applicability of the cost function in practical applications.

The dynamic cost function can be formalized as:

C = w1·T + w2·E + w3·D(1)

where T represents driving time, E represents energy consumption, and D represents driving distance. w1, w2, w3 are the dynamic weight parameters affected by the predicted traffic state. For example, during congestion periods, when the average speed of the road section is less than 20 km/h, the weight value of W1 can be increased from the basic value of 0.3 to 0.5–0.6, and when the vehicle is driving at low speed (10–30 km/h), the energy consumption will be 30–50% higher than that of the economic speed (60–80 km/h), and the weight of W2 can be increased from 0.4 to 0.5–0.55. However, at present, there are no clear standards for how much weight value to adjust in which traffic state and how to determine the priority of weight adjustment in different scenarios. This flexibility in design allows for real-time adaptation but requires precise configuration of weight parameters and balancing multiple goals based on specific scenarios (e.g., EV logistics or urban navigation), and clear dynamic adjustment rules for weights are key to achieving this goal [56].

#### 4.1.3. Path Search Algorithm Overview

The efficiency of path search algorithms in dynamic environments directly impacts the effectiveness of energy-efficient route planning. Dai et al. proposed the Parallel Traffic Condition-Driven Path Planning (PARP) model, which dynamically updates weights and quickly adapts to traffic changes, though its computational load remains substantial [57]. Enhancing the computational efficiency of such algorithms remains a challenge. Moreover, genetic algorithms have shown promise in global searches for multi-objective optimization, particularly in balancing energy consumption and travel time [48], but efficiently finding the optimal path in complex traffic networks still requires further work.

The A* algorithm is widely used in real-time navigation due to its heuristic search properties, with its performance depending on the design of the heuristic function h(n), which can be optimized using traffic predictions [58,59]. Genetic algorithms excel in multi-objective optimization, particularly in balancing energy consumption, time, and distance [48]. Wu et al., based on dynamic nonlinear model predictive control, proposed a smart transportation system path planning method that emphasizes the role of prediction data in enhancing algorithm efficiency [10]. However, the Dijkstra algorithm’s performance in dynamic environments is limited by frequent weight updates, and therefore needs to integrate prediction modules to reduce recalculation frequency.

Collectively, the prediction module provides traffic status forecasts, the cost function guides the path search algorithm, and the combined system ultimately achieves energy-saving goals. The selection of algorithms must balance real-time requirements and solution quality, with A* suitable for rapid responses and genetic algorithms more appropriate for global optimization [48,57].

### 4.2. Review of Classical Path Search Algorithms

Pathfinding algorithms are critical to energy-efficient route planning, as they directly shape how predictive outcomes are translated into actionable decisions for optimizing energy consumption, travel time, and carbon emissions. With solid theoretical foundations and broad applicability, classical algorithms remain highly relevant—even in dynamic traffic environments. The A* algorithm is valued for its efficiency, Dijkstra’s algorithm guarantees global optimality, and multi-objective genetic algorithms (MOGAs) excel at balancing competing objectives. Through an analysis of their principles, applications, and limitations—along with insights from recent research—it becomes evident that current methods face a significant trade-off between real-time performance and global optimization. This highlights the importance of exploring hybrid strategies to advance energy efficiency.

#### 4.2.1. A* Algorithm

A* is a best-first heuristic search that combines the cumulative cost from the start node to a given node g(n) and an estimated cost from that node to the goal h(n) into a single priority function f(n) = g(n) + h(n). Here, h(n) may be computed using Euclidean distance, Manhattan distance, Chebyshev distance, or even domain-specific estimators, provided it never overestimates the true cost (admissibility) and ideally remains consistent (monotonicity). By directing the search toward the goal, A* can reduce node expansions by roughly 30–50% compared with uninformed approaches such as Dijkstra’s algorithm, thereby significantly lowering computation time in real-time applications.

In dynamic routing scenarios, A* has proven remarkably adaptable. For electric-vehicle path planning, Sebai et al. integrated traffic-flow predictions and real-time incident data into both the cost and heuristic models [8]; against Dijkstra and D* Lite benchmarks, their method achieved over 20% reductions in both energy consumption and travel time. Likewise, Gan et al. enhanced inland-vessel routing by embedding a safety-potential field into the heuristic function, which lowered collision-risk metrics by 35% while maintaining efficient voyage times [16]. More recently, Chen et al. demonstrated that incorporating live traffic-signal status into A*’s evaluation can help vehicles avoid red-light delays, cutting average journey times by 15% [48].

Despite these advancements, the performance of the A* algorithm remains profoundly contingent on the caliber of its heuristic function. Should the heuristic h(n) violate the principles of admissibility or consistency, the algorithm may be compelled to re-expand nodes, precipitating a dramatic surge in search costs. Ben Abbes et al. underscore that excessively simplistic heuristic strategies within complex network topologies are prone to yielding suboptimal routes, while high-frequency cost updates in large-scale graphs can render real-time operations impracticable [49]. As such, although A* excels in moderately complex, latency-sensitive tasks like urban navigation, it is not the optimal choice when stringent global optimality or extreme scalability is demanded. In such scenarios, incremental planning algorithms such as D* and LPA*, along with sampling-based methods like RRT* and PRM*, tend to offer more robust performance guarantees.

#### 4.2.2. Dijkstra Algorithm

Dijkstra’s algorithm adopts a greedy strategy, expanding the search step-by-step to ensure the globally shortest path in graphs without negative weights. Although its time complexity is O(V^2^), it can be optimized to O((V + E) log V) using priority queues, making it well-suited for static networks due to its simplicity and optimality. Ben Abbes et al. applied it to electric vehicle routing by integrating energy models and charging station locations, resulting in optimized path selection [49]. Asna et al. also confirmed its reliability in fast-charging station planning in the UAE, especially under static conditions [60].

However, dynamic traffic environments expose Dijkstra’s limitations. Bac et al. noted that each traffic state change (e.g., congestion or time window adjustments) requires recomputation of the entire network, significantly reducing efficiency, especially in scenarios involving partial charging or large-scale networks [61]. Chen et al. attempted to alleviate this burden using traffic flow prediction, yet the computational load of global search remains a challenge [48]. In my view, Dijkstra is more appropriate for relatively stable conditions and needs to be augmented with predictive techniques when applied to real-time dynamic settings.

#### 4.2.3. Multi-Objective Genetic Algorithm (MOGA)

Multi-objective genetic algorithms (MOGAs) have emerged as a unifying framework for reconciling the competing demands of energy consumption, travel time, emissions and safety in intelligent transportation systems. Rather than a mere collection of evolutionary operators, MOGA serves as a narrative thread: it weaves together disparate studies into a coherent exploration of trade-offs, even as it confronts the dual challenges of translating Pareto-optimal solutions into real-world practice and meshing time-intensive offline search with the urgency of live operations.

Early applications of MOGA in vehicle scheduling illustrate both its promise and its limitations. For instance, Zhao et al. integrate short-term traffic-flow forecasts directly into the algorithm’s fitness evaluations, cutting energy use by 12 percent at the cost of only a five-minute delay [29,62,63]. Their work exemplifies prediction-informed evolution, yet stops short of addressing the leap from simulation to live fleet dispatch. Ma et al. extend the approach to highway work zones, optimizing safety margins alongside energy metrics [64]; however, their scenarios remain geographically narrow, leaving open the question of network-wide applicability [13].

Beyond routing, MOGA’s versatility shows in infrastructure design problems such as charging-station placement and electric multiple-unit energy management. Asna et al. [60] and Fischer et al. [65] employ Pareto fronts to illuminate the trade-offs among cost, demand and grid stability, yet seldom reconnect those insights to the operational algorithms that handle real-time rerouting or dispatch. Aghili’s case study in Isfahan begins to bridge this gap by jointly considering station siting and fleet routing, though it still leaves unanswered how fluctuating ridership patterns might dynamically reshape the Pareto landscape [66].

Underneath MOGA’s broad applicability lie significant computational hurdles. Its native ability to handle four or more objectives distinguishes it from scalarization-based methods, but at the price of heavy fitness-evaluation workloads [4,67]. Attempts to streamline the search—whether by task slicing and cache optimization [68], surrogate models that approximate expensive evaluations [69], or federated multi-agent architectures that distribute computation to edge nodes [70]—offer incremental relief but often introduce new challenges, from approximation error to data-heterogeneity and convergence consistency.

Two overarching research gaps stand out. First, no existing pipeline convincingly delivers city-scale MOGA solutions in sub-minute times under live traffic conditions: warm-starts with A* or Dijkstra heuristic searches help, but convergence criteria remain ad hoc rather than principled. Second, studies tend to isolate a single planning layer—dispatch, infrastructure design or traffic prediction—whereas real urban mobility requires an end-to-end, dynamically coordinated ecosystem. The next frontier lies in architecting a modular, layered framework: fast heuristics would seed solutions in real time, genetic search would refine long-term strategies, and surrogate models would prevent staleness. Embedding MOGA as an evolving, interactive component—rather than treating it as a standalone black box—promises to unlock both its academic potential and its practical impact.

### 4.3. Integration Mode Comparison from Literature: A Critical Analysis

This section provides a comprehensive comparison of integration modes, emphasizing the importance of linking theoretical frameworks to practical applications in route planning systems. By analyzing relevant case studies, this section not only examines existing literature but also critiques the strengths, limitations, and potential improvements of different integration strategies. Through a synthesis of previous work, we aim to identify gaps in current methodologies and propose pathways for advancing research in this field.

#### 4.3.1. “Prediction → A” Fast Response Mode*

The “Prediction → A*” fast response mode, a commonly employed strategy in real-time traffic management, leverages real-time traffic predictions to dynamically adjust routing decisions using the A* algorithm. This model is frequently applied in scenarios where immediate adjustments to traffic conditions are required, such as real-time navigation. For instance, Sebai et al. in Optimal Electric Vehicles Route Planning with Traffic Flow Prediction and Real-Time Traffic Incidents demonstrate the practical application of this model in adaptive routing for electric vehicles [8].

Workflow: The model operates by first predicting traffic flow and subsequently updating the graph weights accordingly. Using the updated graph, the A* algorithm identifies a new optimal path.

Critical Analysis: The A* algorithm, while efficient, faces inherent limitations. One of the key concerns is its susceptibility to converging on local optima, especially in complex and highly dynamic environments. The real-time nature of this model makes it computationally attractive; however, this speed comes at the expense of solution quality. The trade-off between computation time and solution quality is an ongoing challenge in real-time systems. While the model excels in scenarios demanding quick decisions, its ability to consistently provide the best possible route in complex traffic conditions remains an area for improvement.

Contribution: This approach is best suited for applications requiring rapid response times, such as GPS navigation and real-time route adjustments for electric vehicles. However, future work could explore hybrid models that combine the speed of A* with global optimization methods to address its limitations in complex scenarios.

#### 4.3.2. “Prediction → Genetic Optimization” Global Optimal Mode

In contrast, the “Prediction → Genetic Optimization” model seeks to optimize long-term route planning by utilizing traffic flow predictions as input to a genetic algorithm, which evolves potential solutions over time. Zhao et al. in Path Planning Based on Traffic Flow Prediction for Vehicle Scheduling provide an in-depth exploration of this model, applying it to vehicle scheduling for large-scale logistics [29,62,63].

Workflow: This model begins by predicting long-term traffic trends and utilizes a genetic algorithm to optimize solutions. The algorithm iteratively evolves a population of routes, optimizing multiple objectives such as energy consumption, travel time, and traffic congestion.

Critical Analysis: While this model excels in optimizing multi-objective functions and providing global optimal solutions, its major drawback is the significant computational overhead required. Genetic algorithms are inherently slower than methods like A*, and this limits their practical application in real-time scenarios. The time required for genetic optimization to converge on a global optimum makes it impractical for fast-response applications. Furthermore, the complexity of multi-objective optimization introduces additional challenges in balancing conflicting goals, such as minimizing energy consumption while reducing travel time.

Contribution: This approach is best suited for scenarios requiring long-term planning, such as fleet management and large-scale logistics optimization. Future research could explore hybrid methods that incorporate genetic algorithms for offline optimization while using faster, real-time models for immediate route adjustments.

#### 4.3.3. Comparative Theoretical Analysis of Methods

The comparison of the two integration modes—“Prediction → A*” and “Prediction → Genetic Optimization”—is summarized in Table 2. This table synthesizes findings from existing studies, providing a clearer understanding of the computational efficiency, solution quality, and applicable scenarios for each mode.

Critical Insights: The “Prediction → A*” mode is highly efficient but sacrifices solution quality in complex environments, making it ideal for real-time navigation where speed is paramount. However, it is clear that the application of A* is limited in scenarios that require more than quick fixes—complex traffic conditions demand methods that can optimize for global solutions. In contrast, the “Prediction → Genetic Optimization” mode offers superior global optimization capabilities, making it suitable for complex, multi-objective problems. However, its computational expense makes it unsuitable for real-time applications where rapid decisions are essential.

Contributions to the Field: This section highlights a critical gap in current research—the need for hybrid approaches that combine the strengths of both modes. Real-time applications would benefit from models that integrate the speed of the A* algorithm with the optimality of genetic optimization. Further exploration of hybrid models, such as combining A* with machine learning techniques for global optimization or integrating genetic algorithms with faster heuristic methods, could provide a promising avenue for future work.

Conclusion and Recommendations: The integration mode chosen must align with the application’s requirements. For real-time scenarios, the “Prediction → A*” fast response mode remains the most practical due to its computational efficiency. For long-term planning, especially in logistics or fleet management, the “Prediction → Genetic Optimization” model is preferred, though it requires careful consideration of its computational demands. Future work should focus on developing hybrid models that can leverage the advantages of both approaches to achieve real-time performance while maintaining global optimality.

## 5. Research Gaps, Challenges, and Future Directions

The interdisciplinary field of energy-efficient route planning and traffic flow prediction has experienced rapid advancement in recent years. However, limitations in data quality, algorithmic efficiency, technological integration, and personalized demand remain substantial barriers to large-scale real-world deployment. This section provides an in-depth discussion of these critical issues, evaluates current research limitations, and synthesizes insights from recent literature to propose forward-looking recommendations aimed at enhancing the effectiveness and sustainability of intelligent transportation systems.

### 5.1. Data Dimension: Quality, Coverage, and Privacy Compliance

High-quality data constitute the foundation of accurate traffic prediction and route planning. Nevertheless, current datasets exhibit significant deficiencies that impair model generalizability and performance [71,72,73]. Abduljabbar et al. [19] and Afandizadeh et al. [2] highlighted considerable spatiotemporal gaps in existing traffic datasets, especially under rural road conditions or extreme weather events, where noise and missing values are prevalent, leading to reduced prediction accuracy. For instance, while urban arterial roads are well-represented in datasets, data scarcity in remote areas causes performance imbalances when models are applied to entire road networks [74]. Alsolami et al. further pointed out that technical and regulatory barriers hinder cross-regional data integration, limiting opportunities for global optimization [20].

Privacy concerns further complicate data acquisition. With the enforcement of regulations such as the General Data Protection Regulation (GDPR), the collection of vehicle trajectories and user behavioral data faces strict constraints [27,75,76,77]. While federated learning and differential privacy have been proposed as potential solutions, their application in transportation remains nascent, with data sharing efficiency still limited [32]. Future research should prioritize the development of high-quality heterogeneous datasets that encompass multimodal transport (e.g., walking, public transit, vehicular traffic) and cover both urban and rural areas, while integrating privacy-preserving technologies to ensure regulatory compliance [78]. For example, the federated learning approach proposed by Khatua et al. [27] facilitates cross-national collaborative model training, and the open multimodal datasets advocated by Chen et al. [10,11] offer a solid foundation for energy-efficient planning [79].

### 5.2. Algorithmic Dimension: Trade-Offs in Real-Time Performance, Scalability, and Interpretability

Algorithm design for energy-efficient route planning encounters multiple challenges. Deep learning models such as Long Short-Term Memory (LSTM) networks and Transformers demonstrate superior accuracy [19,34,80], but their high computational cost limits their suitability for real-time applications. In electric vehicle navigation, where traffic conditions change rapidly, lightweight models such as Convolutional Neural Networks (CNNs) respond quickly but often sacrifice accuracy [43]. Many existing algorithms are designed for small-scale networks; when scaled to interregional networks or fleet-level dispatching, computational efficiency and memory requirements become significant bottlenecks [12,57,81].

The “black-box” nature of AI models undermines trust in critical applications such as traffic control and autonomous driving [50]. Insufficient interpretability not only impedes real-world deployment but also restricts the optimization of decision-making processes [82]. Hybrid frameworks may offer a viable solution—for instance, integrating the transparency of A* algorithms with the predictive power of deep learning [8,58], or leveraging edge computing to enhance real-time responsiveness [10,11]. Advancements in scalability, such as the development of distributed algorithms for large-scale networks [12,57], could significantly enhance the practical utility of energy-efficient planning.

### 5.3. Integration of Emerging Technologies: IoT/5G, Vehicular Networks, and Digital Twins

The Internet of Things (IoT) and 5G technologies provide massive real-time data and low-latency communication capabilities [74,83,84,85]. Vehicular networks (V2X) enable cooperative sensing among vehicles for optimized routing [48], while digital twin systems offer virtual simulations that enhance prediction accuracy [31,86]. Despite their promise, the full potential of these technologies has yet to be realized. While IoT and 5G contribute rich data, efficient processing and cybersecurity remain unresolved challenges [32]. Vehicular networks face privacy issues and a lack of interoperability due to slow progress in standardizing communication protocols [27]. Software-defined vehicular networks, however, offer a flexible approach to optimize traffic efficiency through data-driven V2X coordination [87], supporting real-time path planning in frameworks like “Prediction → A*.”

Digital twins, although promising, entail high computational costs for accurate modeling [31]. For example, real-time construction of a digital twin for urban traffic requires extensive sensor input and physical modeling, which is currently only feasible in pilot-scale implementations [74,88]. Recent advances leverage LiDAR data to create localized digital twins for data-driven traffic simulation, reducing computational overhead while maintaining accuracy [46]. Specific applications include adaptive traffic signal control under limited synchronization conditions, where digital twins enhance prediction robustness for urban networks [89,90], and traffic guidance for autonomous driving, integrating V2X data for energy-efficient routing [24]. These developments align with the article’s proposed integration of prediction and optimization models.

Future directions may include the development of 5G-based vehicular data platforms [74] or the application of digital twins to dynamic traffic forecasting across diverse scenarios, such as extreme weather or peak-hour congestion [31,86]. Addressing security, standardization, and computational challenges will be critical to advancing the intelligence level of energy-efficient planning.

### 5.4. Multimodal Transportation and Personalized Route Planning

Urban mobility is increasingly characterized by truly multimodal journeys—users may cycle to a metro station, ride the train, and then transfer to a bus within a single trip [70,91,92]. Yet most studies remain confined to a single transport mode, lacking the ability to orchestrate real-time data streams, energy-conscious spatial constraints, and regulatory considerations in an integrated fashion [23,93]. For example, in the “bike → metro → bus” scenario, existing algorithms struggle to balance transfer wait times, network-wide energy consumption, and passenger comfort [94,95,96], and they rarely account for legal frameworks or ecological design guidelines that shape urban form and energy potential [93,97].

**Figure 4:**
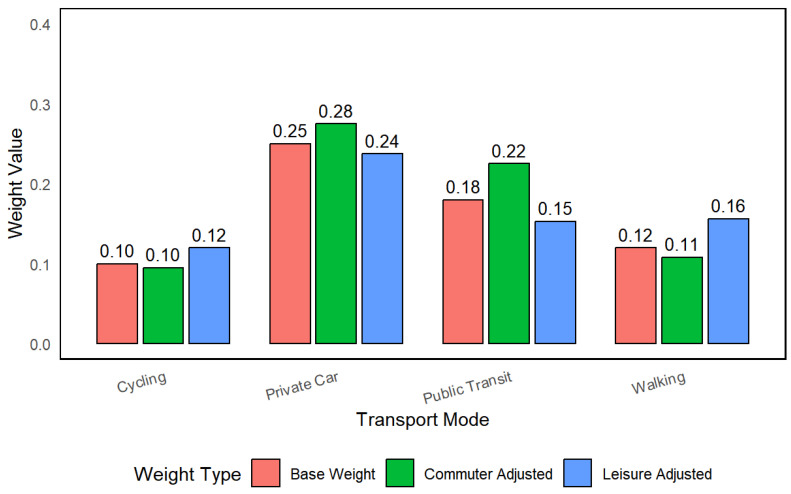
Weight distribution of multimodal modes of transportation.

In multimodal collaborative planning, dynamic cost functions need to be expanded to incorporate mode-specific parameters. For walking segments, comfort metrics such as pavement evenness and shade coverage (weight w_4_) should be included. Public transit modules should account for headway and number of transfers (weight w_5_), while cycling components need to incorporate slope and dedicated lane length (weight w_6_). The extended cost function can be formalized as follows:

C = w1·T + w2·E + w3·D + w4·Comf_walk + w5·Transit_freq + w6·Cycle_slope(2)

where Comf_walk represents pedestrian comfort scores (1–5), Transit_freq denotes bus headway (minutes), and Cycle_slope indicates average road gradient (%). Empirical data from the Tokyo metropolitan area shows that during morning peak hours (7:30–9:00), each 1-point decrease in walking comfort reduces user adoption by 18–22%, necessitating an upward adjustment of w4 from 0.12 to 0.18–0.22 [98], see Figure 4.

Moreover, individual travelers prioritize different objectives—some seek minimum energy use, others shortest travel time or highest comfort—yet general-purpose models lack the dynamic, profile-driven adaptability to satisfy these divergent needs [99,100,101]. To address these gaps, we advocate for a unified multimodal transport-data platform augmented by personalized, reinforcement-learning and real-time optimization modules. For instance:

(1) Dynamic route adaptation: Basso et al. [56] demonstrate how a reinforcement-learning agent can continuously recalibrate route recommendations to optimize both energy use and travel time [102]. For user preferences, a preference matrix P = [p1, p2, p3, p4, p5, p6] can be introduced, where p1–p6 correspond to weights for time, energy, distance, walking comfort, transit convenience, and cycling friendliness (summing to 1). When a user selects comfort-prioritized routes three consecutive times, the system automatically increases p4 by 20% [103].
(2) Real-time synchronization: Chen et al. [48] show that techniques drawn from industrial online scheduling can improve transfer timing and fleet utilization [104,105]. In multimodal scenarios, this can dynamically balance transfer wait times and energy costs—for example, when metro delays exceed 5 min, triggering automatic weight adjustments for bus/cycling alternatives [74,81], see Table 3.

### 5.5. Recommendations: Standardized Evaluation Platforms and Open-Source Toolkits

Imagine a city planner logging into a single portal that instantly benchmarks her new routing algorithm against a standardized metric suite—from base-level travel-time and energy-use indicators [103,106] to passenger-perception scores drawn from a validated survey instrument [107]. Behind the scenes, each experiment also feeds into a prioritization module [108] that ranks proposed network upgrades by cost, social benefit and CO_2_ reduction, and an investment-appraisal dashboard modeled on the Dutch standardized framework [109,110].

To bring this vision to life, we recommend three intertwined pillars:

Unified Evaluation Backbone

Core metrics registry: Adopt a minimal yet extensible set of “must-report” indicators [22,106] covering throughput, delay, energy-use per passenger-km, emissions, and service quality ratings [107].

Pluggable appraisal engines: Embed modules for cost–benefit analysis [109], energy-efficiency utility modeling [103], and multi-criteria ranking [108], so every study yields comparable scores.

Open-Source, Modular Toolkit

Data & model registry: A repository of canonical datasets (urban/rural/highway), reference algorithms (GCNs, A*, RL planners) and pre-built connectors to GIS and traffic simulators, all versioned and containerized [22].

Survey & perception plug-in: Standardized forms and analytics scripts for gathering and integrating commuter feedback [107,111].

Community-Driven Governance & Evolution

Rotating steering group of academics, practitioners, and policymakers to ratify new metrics, datasets, and modules—ensuring the platform reflects emerging needs [22].

Living documentation and hackathons to crowdsource additions, troubleshoot reproducibility gaps, and showcase real-world deployments.

By weaving together decades of methodological advances—from base-level metric standardization and investment evaluation to energy-utility modeling and legal/operational management systems—this ecosystem will transform isolated proofs-of-concept into a shared, evolving infrastructure [22,103,106,109]. Researchers gain immediate comparability; practitioners access turn-key tools; and policymakers receive transparent, data-backed ranking of every proposed improvement. In effect, the field transcends ad hoc experiments and enters a new era of rapid, reproducible innovation and real-world impact [112,113].

## 6. Conclusions

This review comprehensively examined recent advancements in traffic flow prediction and energy-efficient route planning, underscoring the pivotal role of AI technologies in enhancing prediction accuracy and route optimization—particularly through deep learning models. Through a critical comparison with traditional pathfinding algorithms, it was found that while deep learning models excel in accuracy, their limitations in real-time responsiveness and interpretability necessitate integration with conventional methods. Emerging technologies such as IoT, 5G, and digital twins have introduced novel pathways for innovation, although practical deployment remains constrained by technical and regulatory challenges. The unique contribution of this study lies in its proposal of an integrated theoretical framework that emphasizes the importance of data quality, algorithmic efficiency, and personalized demand in cross-domain system design for energy-efficient planning.

Nonetheless, as a literature-based review, the study did not conduct empirical validation or algorithmic benchmarking, which may limit the generalizability of some conclusions, especially in light of regional disparities in transportation systems. Future studies incorporating case-based analysis and quantitative experiments are expected to enhance the credibility of findings. In summary, future research in intelligent transportation should focus on improving data quality and coverage, exploring privacy-preserving technologies for data sharing, developing lightweight and interpretable AI models, and integrating traditional algorithms to improve real-time and global performance. Furthermore, the convergence of IoT, 5G, and digital twins should be advanced to overcome challenges related to security and standardization. Building multimodal and personalized planning systems will address diverse user needs, while standardized evaluation platforms and open-source tools will catalyze technological advancement. These directions will collectively contribute to a high-efficiency, low-emission, and secure future for intelligent transportation systems, aligned with the goals of sustainable urban development.

## Figures and Tables

**Table 1:** Summary of Traffic Flow Prediction Methods: Advantages, Disadvantages, and Applications.

Method Category	Method Name	Advantages	Disadvantages	Applicable Scenarios
Statistical Models	ARIMA and SARIMA	Simple structure, computationally efficient, suitable for stationary time series data [34]	High data stationarity requirement, ineffective for sudden events or short-term fluctuations [36]	Long-term, stable traffic flow prediction [34]
Statistical Models	Kalman Filter	Real-time updates, low latency, effective for handling noisy and uncertain data [38]	Assumes Gaussian noise, not suitable for non-Gaussian or complex dynamic flows [22]	Small-scale real-time traffic monitoring, micro-level dynamic adjustments [22]
Machine Learning Models	Linear Regression & SVR	Easy to understand, SVR effectively handles complex non-linearities [48]	Sensitive to noise, computationally expensive, less accurate with complex data [48]	Medium-term predictions, scenarios with strong linear relationships between features and targets
Machine Learning Models	Random Forest (RF)	Strong non-linear modeling capability, resistant to overfitting, automatic feature selection [23]	High computational cost, especially with large datasets; tree depth and number significantly affect model speed	Multi-feature traffic flow prediction, energy-efficient route planning
Machine Learning Models	XGBoost and LightGBM	Strong predictive power, efficient with large datasets, supports real-time updates [49]	Complex model tuning, highly sensitive to missing values and hyperparameter selection	Large-scale real-time traffic flow prediction, dynamic route adjustments
Deep Learning Models	Convolutional Neural Networks (CNN)	Efficient in extracting spatial features, well-suited for learning road network structures [50]	Limited in capturing temporal dependencies, typically requires integration with models like LSTM [24,25,26,47]	Spatial data analysis in traffic flow prediction, road network modeling
Deep Learning Models	LSTM & GRU	Captures long-term dependencies, effective for dynamic traffic flow prediction [19]	Sensitive to noise, requires large datasets, computationally expensive [19]	Short-term traffic forecasting, spatiotemporal speed prediction
Deep Learning Models	Spatiotemporal GNNs	Models both spatial and temporal dependencies, suitable for large-scale traffic networks [2]	High computational complexity, slower inference speed [2]	Large-scale real-time traffic forecasting, congestion detection
Hybrid and Enhanced Approaches	Wavelet Denoising + XGBoost	Wavelet denoising improves data quality, XGBoost enhances prediction accuracy [20]	High computational overhead, may hinder real-time applications	Traffic data with noise or low signal, improving model performance in noisy datasets
Hybrid and Enhanced Approaches	MLR + LSTM	Combines traditional statistical models with deep learning, capturing both linear and non-linear features [24,25,26,47]	Integration complexity, may require tuning for optimal performance	Traffic prediction with both linear and non-linear dependencies
Hybrid and Enhanced Approaches	Dual Error Model (DEM)	Improves prediction stability and accuracy by integrating model and observational errors [27]	High computational complexity when merging multiple error sources	Complex, uncertain traffic data scenarios, federated learning for traffic prediction

**Table 2:** Comparison of “Prediction → A” and “Prediction → Genetic Optimization” Modes*.

Mode	Computational Efficiency	Solution Quality	Suitable Scenarios	Supporting Literature
Prediction → A*	High	Local Optimum	Real-time navigation, fast response	Sebai et al. (2022); Dai et al. (2021); Jose & Vijula Grace (2022) [8,57,58]
Prediction → Genetic Optimization	Low	Global Optimum	Long-term planning, multi-objective optimization	Zhao et al. (2023); Li et al. (2022); Basso et al. (2022) [12,29,56]

**Table 3:** Literature summary on Energy Optimization and Urban Transportation Systems.

Reference	Core Method/Perspective	Application & Value
Stremke & Koh (2010) [95]	Ecological design strategies for energy-conscious spatial planning	Provides principles for land-use layouts that reduce energy demand and support multimodal hubs
Sachanbińska-Dobrzyńska (2023) [93]	Comparative legal analysis	Highlights Poland/Germany regulatory frameworks to ensure algorithmic planning remains compliant
Stoeglehner & Narodoslawsky (2012) [96]	Strategic planning for energy-optimized urban structures	Austrian case studies illustrating coordination of urban form with district-scale energy systems
Van den Dobbelsteen et al. (2012) [97]	Energy-potential and thermal-mapping techniques	Develops GIS-based maps to identify low-energy corridors and transfer nodes
Jiang et al. (2022) [100]	Flexible job-shop scheduling with transport and deterioration	Introduces dual constraints of travel time and equipment aging, inspiring multimodal scheduling
Oberwinkler & Stundner (2005) [104]	Real-time production optimization	Adapts industrial online scheduling algorithms for dynamic transit vehicle dispatch
Golrezaei et al. (2014) [101]	Real-time optimization of personalized assortments	Validates user-profile-driven decision frameworks in retail, offering insights for transport choices
Diehl (2001) [105]	Online optimization for large-scale nonlinear processes	Offers algorithmic foundations for high-dimensional, constrained real-time optimization
Goldman & Gorham (2006) [83]	Four innovative directions in sustainable urban transport	Proposes macro-strategies (e.g., demand management, technological integration) for multimodal systems
Boschmann & Kwan (2008) [84]	Social sustainability in urban transportation	Emphasizes equity and accessibility metrics to enrich user-satisfaction dimensions
Gudmundsson & Regmi (2017) [85]	Sustainable Urban Transport Index (SUTI)	Constructs a composite indicator for benchmarking multimodal network performance

## Data Availability

Not applicable.

## References

[1] Sayed S.A., Abdel-Hamid Y., Hefny H.A. (2023). Artificial intelligence-based traffic flow prediction: A comprehensive review. J. Electr. Syst. Inf. Technol..

[2] Afandizadeh S., Abdolahi S., Mirzahossein H. (2024). Deep learning algorithms for traffic forecasting: A comprehensive review and comparison with classical ones. J. Adv. Transp..

[3] Mystakidis A., Koukaras P., Tjortjis C. (2025). Advances in traffic congestion prediction: An overview of emerging techniques and methods. Smart Cities.

[4] Gao X., Ci Y., Fai Y.K., Wu L., Li R. (2025). Hybrid traffic flow prediction model for emergency scenarios with scarce historical data. Eng. Appl. Artif. Intell..

[5] Xie Z., Liu Z., Peng Z., Wu W., Zhou B. (2025). Vid2Sim: Realistic and Interactive Simulation from Video for Urban Navigation. arXiv.

[6] He M., Lake P. (2024). Artificial intelligence-based traffic signal control for urban transportation systems. Proceedings of the 2024 IEEE Integrated STEM Education Conference (ISEC).

[7] Li J., Liu Z., Wang X. (2022). Public charging station localization and route planning of electric vehicles considering the operational strategy: A bi-level optimizing approach. Sustain. Cities Soc..

[8] Sebai M., Rejeb L., Denden M.A., Amor Y., Baati L., Said L.B. (2022). Optimal electric vehicles route planning with traffic flow prediction and real-time traffic incidents. Int. J. Electr. Comput. Eng. Res..

[9] Jia S., Peng H., Liu S. (2009). Review of transportation and energy consumption related research. J. Transp. Syst. Eng. Inf. Technol..

[10] Chen S., Wen H., Wu J. (2022). Artificial intelligence based traffic control for edge computing assisted vehicle networks. J. Internet Technol..

[11] Chen X., Liu Y., Zhang J. (2022). Traffic prediction for Internet of Things through support vector regression model. Internet Technol. Lett..

[12] Li B., Dai T., Chen W., Song X., Zang Y., Huang Z., Lin Q., Cai K. (2022). T-PORP: A trusted parallel route planning model on dynamic road networks. IEEE Trans. Intell. Transp. Syst..

[13] Ma W., Chen B., Yu C., Qi X. (2022). Trajectory planning for connected and autonomous vehicles at freeway work zones under mixed traffic environment. Transp. Res. Rec..

[14] Xia D., Zheng L., Tang Y., Cai X., Chen L., Sun D. (2022). Dynamic traffic prediction for urban road network with the interpretable model. Phys. A Stat. Mech. Its Appl..

[15] Lin G., Lin A., Gu D. (2022). Using support vector regression and K-nearest neighbors for short-term traffic flow prediction based on maximal information coefficient. Inf. Sci..

[16] Gan L., Yan Z., Zhang L., Liu K., Zheng Y., Zhou C., Shu Y. (2022). Ship path planning based on safety potential field in inland rivers. Ocean. Eng..

[17] Wu Y., Li S., Zhang Q., Sun-Woo K., Yan L. (2022). Route planning and tracking control of an intelligent automatic unmanned transportation system based on dynamic nonlinear model predictive control. IEEE Trans. Intell. Transp. Syst..

[18] Srivastava A., Singh M., Nandi S. (2025). Support Vector Regression Based Traffic Prediction Machine Learning Model. Proceedings of the 2024 8th International Conference on Computational System and Information Technology for Sustainable Solutions (CSITSS).

[19] Abduljabbar R.L., Dia H., Tsai P.W., Liyanage S. (2021). Short-term traffic forecasting: An LSTM network for spatial-temporal speed prediction. Future Transp..

[20] Alsolami B., Mehmood R., Albeshri A. (2020). Hybrid statistical and machine learning methods for road traffic prediction: A review and tutorial. Smart Infrastructure and Applications: Foundations for Smarter Cities and Societies.

[21] MSK M. (2023). Hybrid long short-term memory deep learning model and Dijkstra’s Algorithm for fastest travel route recommendation considering eco-routing factors. Transp. Lett..

[22] Shi X. Design and Application of Transportation Standardization Management System. Proceedings of the 2018 10th International Conference on Computer and Automation Engineering.

[23] Alfaseeh L., Farooq B. (2020). Multi-factor taxonomy of eco-routing models and future outlook. J. Sens..

[24] Liao X., Leng S., Sun Y., Zhang K., Imran M.A. (2024). A Digital Twin-Based Traffic Guidance Scheme for Autonomous Driving. IEEE Internet Things J..

[25] Ji W., Zhang T., Xu B., He G. (2024). Apple recognition and picking sequence planning for harvesting robot in a complex environment. J. Agric. Eng..

[26] Ji W., He G., Xu B., Zhang H., Yu X. (2024). A New Picking Pattern of a Flexible Three-Fingered End-Effector for Apple Harvesting Robot. Agriculture.

[27] Khatua S., De D., Maji S., Maity S., Nielsen I.E. (2024). A federated learning model for integrating sustainable routing with the Internet of Vehicular Things using genetic algorithm. Decis. Anal. J..

[28] Liu Y., Huo L., Zhang X., Wu J. (2023). A multi-objective resource pre-allocation scheme using SDN for intelligent transportation system. IEEE Trans. Intell. Transp. Syst..

[29] Zhao Y., Mo L., Liu J. (2023). Path planning based on traffic flow prediction for vehicle scheduling. Proceedings of the 2023 IEEE/CIC International Conference on Communications in China (ICCC).

[30] Sheng P., He Y., Guo X. (2017). The impact of urbanization on energy consumption and efficiency. Energy Environ..

[31] Chandra S. (2025). Sustainable Urban Mobility and Smart Traffic Management: Balancing Optimization With Environmental Sustainability Goals. Machine Learning and Robotics in Urban Planning and Management.

[32] Madupuri R.P., Sobin C.C., Enduri M.K., Anamalamudi S. (2024). Swarm Intelligence in IoT and Edge Computing. Swarm Intelligence.

[33] Wang Z., Wang S. (2022). Real-time dynamic route optimization based on predictive control principle. IEEE Access.

[34] Dubey A.K., Kumar A., García-Díaz V., Sharma A.K., Kanhaiya K. (2021). Study and analysis of SARIMA and LSTM in forecasting time series data. Sustain. Energy Technol. Assess..

[35] Cheng J., Sun J., Shi L., Dai C. (2024). An effective method fusing electronic nose and fluorescence hyperspectral imaging for the detection of pork freshness. Food Biosci..

[36] Sirisha U.M., Belavagi M.C., Attigeri G. (2022). Profit prediction using ARIMA, SARIMA and LSTM models in time series forecasting: A comparison. IEEE Access.

[37] Estil-Les M.A.D.C., Mangini A.M., Roccotelli M., Fanti M.P. (2025). Electric Vehicle Routing Optimization for Postal Delivery and Waste Collection in Smart Cities. IEEE Trans. Intell. Transp. Syst..

[38] Chen R., Chen B. (2024). Traffic State Estimation Using Basic Safety Messages Based on Kalman Filter Technique. Proceedings of the 2024 IEEE 6th International Conference on Civil Aviation Safety and Information Technology (ICCASIT).

[39] Lin B., Du Z. (2015). How China’s urbanization impacts transport energy consumption in the face of income disparity. Renew. Sustain. Energy Rev..

[40] Sun B., Sun T., Jiao P. (2021). Spatio-temporal segmented traffic flow prediction with ANPRS data based on improved XGBoost. J. Adv. Transp..

[41] Xue Y., Jiang H. (2023). Monitoring of chlorpyrifos residues in corn oil based on raman spectral deep-learning model. Foods.

[42] Deng J., Chen Z., Jiang H., Chen Q. (2025). High-precision detection of dibutyl hydroxytoluene in edible oil via convolutional autoencoder compressed fourier-transform near-infrared spectroscopy. Food Control.

[43] Pirani M., Thakkar P., Jivrani P., Bohara M.H., Garg D. A comparative analysis of ARIMA, GRU, LSTM and BiLSTM on financial time series forecasting. Proceedings of the 2022 IEEE International Conference on Distributed Computing and Electrical Circuits and Electronics (ICDCECE).

[44] Moradi N., Boroujeni N.M. (2025). Prize-collecting Electric Vehicle routing model for parcel delivery problem. Expert Syst. Appl..

[45] Zhao Y., Xu S., Duan H. (2024). HGNN−BRFE: Heterogeneous Graph Neural Network Model Based on Region Feature Extraction. Electronics.

[46] Wibisana M.I. (2024). Leveraging LiDAR Data and Local Digital Twins Framework for Data-Driven Traffic Simulation. Master’s Thesis.

[47] Alsuwat H., Alsuwat E. (2025). Energy-aware and efficient cluster head selection and routing in wireless sensor networks using improved artificial bee Colony algorithm. Peer-To-Peer Netw. Appl..

[48] Aung N., Dhelim S., Chen L., Lakas A., Zhang W., Ning H., Chaib S., Kechadi M.T. (2023). VeSoNet: Traffic-aware content caching for vehicular social networks using deep reinforcement learning. IEEE Trans. Intell. Transp. Syst..

[49] Ben Abbes S., Rejeb L., Baati L. (2022). Route planning for electric vehicles. IET Intell. Transp. Syst..

[50] D’Angelo G., Palmieri F. (2021). Network traffic classification using deep convolutional recurrent autoencoder neural networks for spatial–temporal features extraction. J. Netw. Comput. Appl..

[51] Gao Z., Wu Z., Hao W., Long K., Byon Y.J., Long K. (2021). Optimal trajectory planning of connected and automated vehicles at on-ramp merging area. IEEE Trans. Intell. Transp. Syst..

[52] Reza I., Ratrout N.T., Rahman S.M. (2021). Artificial intelligence-based protocol for macroscopic traffic simulation model development. Arab. J. Sci. Eng..

[53] Al Duhayyim M., Albraikan A.A., Al-Wesabi F.N., Burbur H.M., Alamgeer M., Hilal A.M., Hamza M.A., Rizwanullah M. (2022). Modeling of artificial intelligence based traffic flow prediction with weather conditions. Comput. Mater. Contin..

[54] Kadkhodayi A., Jabeli M., Aghdam H., Mirbakhsh S. (2023). Artificial intelligence-based real-time traffic management. J. Electr. Electron. Eng..

[55] Sun J., Cheng J., Xu M., Yao K. (2024). A method for freshness detection of pork using two-dimensional correlation spectroscopy images combined with dual-branch deep learning. J. Food Compos. Anal..

[56] Basso R., Kulcsár B., Sanchez-Diaz I., Qu X. (2022). Dynamic stochastic electric vehicle routing with safe reinforcement learning. Transp. Res. Part E Logist. Transp. Rev..

[57] Dai T., Li B., Yu Z., Tong X., Chen M., Chen G. (2021). PARP: A parallel traffic condition driven route planning model on dynamic road networks. ACM Trans. Intell. Syst. Technol. (TIST).

[58] Jose C., Vijula Grace K.S. (2022). Optimization based routing model for the dynamic path planning of emergency vehicles. Evol. Intell..

[59] Peng N., Xi Y., Rao J., Ma X., Ren F. (2021). Urban multiple route planning model using dynamic programming in reinforcement learning. IEEE Trans. Intell. Transp. Syst..

[60] Asna M., Shareef H., Achikkulath P., Mokhlis H., Errouissi R., Wahyudie A. (2021). Analysis of an optimal planning model for electric vehicle fast-charging stations in Al Ain City, United Arab Emirates. IEEE Access.

[61] Bac U., Erdem M. (2021). Optimization of electric vehicle recharge schedule and routing problem with time windows and partial recharge: A comparative study for an urban logistics fleet. Sustain. Cities Soc..

[62] Zhang Y., Cao W., Zhao H., Gao S. (2023). Route planning algorithm based on dynamic programming for electric vehicles delivering electric power to a region isolated from power grid. Artif. Life Robot..

[63] Zhao N., Zhang F., Yang Y., Coskun S., Lin X., Hu X. (2023). Dynamic traffic prediction-based energy management of connected plug-in hybrid electric vehicles with long short-term state of charge planning. IEEE Trans. Veh. Technol..

[64] Zhang S., James J.Q. (2021). Electric vehicle dynamic wireless charging system: Optimal placement and vehicle-to-grid scheduling. IEEE Internet Things J..

[65] Fischer S., Hermán B., Sysyn M., Kurhan D., Szürke S.K. (2025). Quantitative analysis and optimization of energy efficiency in electric multiple units. Facta Univ. Ser. Mech. Eng..

[66] Akbari F., Mahpour A., Ahadi M.R. (2020). Evaluation of Energy Consumption and CO_2_ Emission Reduction Policies for Urban Transport with System Dynamics Approach. Environ. Model. Assess..

[67] Mu C., Du L., Zhao X. (2021). Event triggered rolling horizon based systematical trajectory planning for merging platoons at mainline-ramp intersection. Transp. Res. Part C Emerg. Technol..

[68] Sifeng Z., Zhaowei S., Hai Z., Rui Q. (2025). Efficient slicing scheme and cache optimization strategy for structured dependent tasks in intelligent transportation scenarios. Ad Hoc Netw..

[69] Xiong S., Xie Y., Zou C., Mao Y., Cao Y. (2024). Optimised design of cross-shaft parameters based on response surface optimization model with MOGA. Int. J. Wirel. Mob. Comput..

[70] Mamond A.W., Kundroo M., Yoo S.E., Kim S., Kim T. (2025). FLDQN: Cooperative Multi-Agent Federated Reinforcement Learning for Solving Travel Time Minimization Problems in Dynamic Environments Using SUMO Simulation. Sensors.

[71] Sattarzadeh A.R., Kutadinata R.J., Pathirana P.N., Huynh V.T. (2025). A novel hybrid deep learning model with ARIMA Conv-LSTM networks and shuffle attention layer for short-term traffic flow prediction. Transp. A Transp. Sci..

[72] Ding Y., Zeng R., Jiang H., Guan X., Jiang Q., Song Z. (2024). Classification of tea quality grades based on hyperspectral imaging spatial information and optimization models. J. Food Meas. Charact..

[73] Xiao L., Li S., Wen Q., Liang X., Li Y., Wang W., Fu Y. (2025). Load balancing routing algorithm of industrial wireless network for digital twin. Comput. Netw..

[74] Chen W., Liu B., Han W., Li G., Song B. (2024). Dynamic path planning based on traffic flow prediction and traffic light status. International Conference on Algorithms and Architectures for Parallel Processing, Proceedings of the 23rd International Conference on Algorithms and Architectures for Parallel Processing, Tianjin, China, 20–22 October 2023.

[75] Kolankeh A.K. (2025). A Hybrid Optimization Algorithm for Large-Scale Combinatorial Problems in Cloud Computing Environments. ALCOM J. Algorithm Comput..

[76] Zelani S.K., Babu D.N., Surendra D., Rahman M.Z.U. (2025). Energy-Efficient Ant Routing Algorithm for Optimized Path Selection Network Longevity in Wireless Sensor Networks. Telecommun. Radio Eng..

[77] Ding C., Wang L., Chen L.S.X. (2023). A blockchain-based wide-area agricultural machinery resource scheduling system. Appl. Eng. Agric..

[78] Lyridis D.V. (2021). An improved ant colony optimization algorithm for unmanned surface vehicle local path planning with multi-modality constraints. Ocean Eng..

[79] Schoenberg S., Dressler F. (2022). Reducing waiting times at charging stations with adaptive electric vehicle route planning. IEEE Trans. Intell. Veh..

[80] ArunKumar K.E., Kalaga D.V., Kumar C.M.S., Kawaji M., Brenza T.M. (2022). Comparative analysis of Gated Recurrent Units (GRU), long Short-Term memory (LSTM) cells, autoregressive Integrated moving average (ARIMA), seasonal autoregressive Integrated moving average (SARIMA) for forecasting COVID-19 trends. Alex. Eng. J..

[81] Tiwari A.K., Pandey R.K., Singh S., Tiwari G., Kumar A., Mishra P. (2024). Artificial intelligence-based smart traffic control system. Proceedings of the International Conference on Innovations in Data Analytics.

[82] Knigge D.M., Romero D.W., Gu A., Gavves E., Bekkers E.J., Tomczak J.M., Hoogendoorn M., Sonke J.J. (2023). Modelling long range dependencies in nd: From task-specific to a general purpose CNN. arXiv.

[83] Goldman T., Gorham R. (2006). Sustainable urban transport: Four innovative directions. Technol. Soc..

[84] Boschmann E.E., Kwan M.P. (2008). Toward socially sustainable urban transportation: Progress and potentials. Int. J. Sustain. Transp..

[85] Gudmundsson H., Regmi M.B. (2017). Developing the sustainable urban transport index. Transp. Sustain. Dev. Goals.

[86] Irfan M.S., Dasgupta S., Rahman M. (2024). Towards transportation digital twin systems for traffic safety and mobility: A review. IEEE Internet Things J..

[87] Shahriar M.S., Subramaniam S., Matsuura M., Hasegawa H., Lin S.C. (2024). Digital Twin Enabled Data-Driven Approach for Traffic Efficiency and Software-Defined Vehicular Network Optimization. Proceedings of the 2024 IEEE 100th Vehicular Technology Conference (VTC2024-Fall).

[88] Szczepanski R., Tarczewski T., Erwinski K. (2022). Energy efficient local path planning algorithm based on predictive artificial potential field. IEEE Access.

[89] Mirbakhsh S. (2023). Artificial intelligence-based real-time traffic management review article. J. Electr. Electron. Eng..

[90] Zhu H., Sun F., Tang K., Wu H., Feng J., Tang Z. (2024). Digital Twin-Enhanced Adaptive Traffic Signal Framework under Limited Synchronization Conditions. Sustainability.

[91] Franzin A., Stützle T. (2025). A causal framework for stochastic local search optimization algorithms. Comput. Oper. Res..

[92] Duan J., Cao P., Shu T., Zhou B., Xue L., Yang L. (2023). Effects of cellulosic carbon addition on nitrogen removal from simulated dry land drainage, and its environmental effects. Agronomy.

[93] Sachanbińska-Dobrzyńska O. (2023). A legal framework for energy-conscious urban planning in Poland and Germany. Energies.

[94] Agrahari A., Dhabu M.M., Deshpande P.S., Tiwari A., Baig M.A., Sawarkar A.D. (2024). Artificial intelligence-based adaptive traffic signal control system: A comprehensive review. Electronics.

[95] Stremke S., Koh J. (2010). Ecological concepts and strategies with relevance to energy-conscious spatial planning and design. Environ. Plan. B Plan. Des..

[96] Stoeglehner G., Narodoslawsky M. (2012). Energy-conscious planning practice in Austria: Strategic planning for energy-optimized urban structures. Sustainable Energy Landscapes: Designing, Planning and Development.

[97] Van den Dobbelsteen A., Broersma S., Fremouw M., Stremke S., van den Dobbelsteen A. (2012). Energy potential mapping and heat mapping: Prerequisite for energy-conscious planning and design. Sustainable Energy Landscapes; Designing, Planning and Development.

[98] Chen P.W. (2023). Research on the Scheme Comparison of Xuzhou Urban Rail Transit Network Based on Network Form. J. Phys. Conf. Ser..

[99] Liang G., Kintak U., Ning X., Tiwari P., Nowaczyk S., Kumar N. (2023). Semantics-aware dynamic graph convolutional network for traffic flow forecasting. IEEE Trans. Veh. Technol..

[100] Jiang T., Zhu H., Liu L., Gong Q. (2022). Energy-conscious flexible job shop scheduling problem considering transportation time and deterioration effect simultaneously. Sustain. Comput. Inform. Syst..

[101] Golrezaei N., Nazerzadeh H., Rusmevichientong P. (2014). Real-time optimization of personalized assortments. Manag. Sci..

[102] Lee H., Kim K., Kim N., Cha S.W. (2022). Energy efficient speed planning of electric vehicles for car-following scenario using model-based reinforcement learning. Appl. Energy.

[103] Sears J., Glitman K., Fanslow G. (2013). Measure for Measure: Energy Utility Model for Standardized Evaluation of Transportation Efficiency Measures. Transp. Res. Rec..

[104] Oberwinkler C., Stundner M. (2005). From real-time data to production optimization. SPE Prod. Facil..

[105] Diehl M. (2001). Real-Time Optimization for Large Scale Nonlinear Processes. Ph.D. Thesis.

[106] Marsh K.D. (1995). Establishing a Standardized Set of Base-Level Transportation Metrics.

[107] Verma V.K. (2025). Standardization of Commuter Perception Survey to Measure Transit Service Quality–An Indian Study. Transp. Res. Procedia.

[108] Turochy R.E. (2001). Prioritizing proposed transportation improvements: Methods, evaluation, and research needs. Transp. Res. Rec..

[109] Annema J.A., Koopmans C., Van Wee B. (2007). Evaluating transport infrastructure investments: The Dutch experience with a standardized approach. Transp. Rev..

[110] Chen L., Xie C., Ma D., Yang Y., Li Y. (2025). A short-term traffic flow prediction model for road networks using inverse isochrones to determine dynamic spatiotemporal correlation ranges. Phys. A Stat. Mech. Its Appl..

[111] Chatterjee A., Paul S.K., Mukhopadhyay S. (2025). Sustainable Urban Transportation: A Standardized Solution or Mixed Model System?. Sustainability and Urban Quality of Life.

[112] Wu X., Wang Y., Wu B., Sun J. (2024). Classification of fritillaria using a portable near-infrared spectrometer and fuzzy generalized singular value decomposition. Ind. Crops Prod..

[113] Gönlügür E. (2025). Hope, Indignation, Nostalgia: The Emotional Navigation of Urban Modernity in Post-War Istanbul. Capitalist Cold.

